# Density and size of lymphoid follicles are useful clues in differentiating primary intestinal follicular lymphoma from intestinal reactive lymphoid hyperplasia

**DOI:** 10.1186/s13000-020-00991-3

**Published:** 2020-07-07

**Authors:** Hsin-Ni Li, Ren Ching Wang, Jun-Peng Chen, Sheng-Tsung Chang, Shih-Sung Chuang

**Affiliations:** 1 Department of Pathology and Laboratory Medicine, Taichung Veterans General Hospital, Taichung, Taiwan; 2 Department of Nursing, HungKuang University, Taichung, Taiwan; 3 Biostatistics Task Force, Taichung Veterans General Hospital, Taichung, Taiwan; 4 Department of Pathology, Chi-Mei Medical Center, 901 Chung-Hwa Road, Yong-Kang District, Tainan, 71004 Taiwan; 5 Department of Nursing, National Tainan Institute of Nursing, Tainan, Taiwan; 6 Department of Pathology, School of Medicine, College of Medicine, National Taiwan University, Taipei, Taiwan

**Keywords:** Digital pathology, Duodenal-type follicular lymphoma, Follicular lymphoma, Intestinal lymphoma, Lymphoid hyperplasia, Whole slide imaging

## Abstract

**Background:**

Primary intestinal follicular lymphoma (PI-FL) is a rare and indolent lymphoma and is challenging for diagnosis with endoscopic biopsy specimens. Whole slide imaging (WSI) has been increasingly used for assisting pathologic diagnosis, but not for lymphoma yet, probably because there are usually too many immunostained sections in a single case. In this study we attempted to identify morphological clues of PI-FL in the endoscopic biopsy specimens by measuring various parameters using WSI.

**Methods:**

We retrospectively investigated 21 PI-FL cases, and scanned the HE sections from 17 of these cases with endoscopic biopsy specimens. Sections from 17 intestinal biopsies showing reactive lymphoid hyperplasia were scanned for comparison. The density and diameter of lymphoid follicles and the shortest distance of these follicles to the surface epithelia were measured on WSI. Comparisons of the aforementioned parameters were made between the neoplastic and reactive follicles.

**Results:**

The density of follicles was significantly higher in PI-FL than that of reactive hyperplasia (median 0.5 vs. 0.2/mm^2^; *p* < 0.01). Furthermore, the neoplastic follicles were significantly larger (median diameter 756.9 vs. 479.7 μm; *p* < 0.01). The shortest distance of follicles to the surface epithelia tended to be closer in PI-FL (104.7 vs. 177.8 μm, *p* = 0.056), but not statistically significant.

**Conclusions:**

In this study we found that in PI-FL the density and diameter of lymphoid follicles as measured from WSI were significantly different from that of intestinal reactive lymphoid hyperplasia. When facing the diagnostic challenge between these two entities in routine practice, pathologists might be alerted by these morphological clues and request for immunohistochemistry for differential diagnosis.

## Background

Whole slide imaging (WSI), the scanning of conventional glass slides as digital files, has been increasingly employed by pathologists for assisting diagnosis, education, and research purposes. Recently several studies have validated the accuracy of WSI in pathological diagnosis, suggesting that WSI can be used for routine practice, which has been realized in a Dutch academic pathology laboratory where the pathological diagnosis is fully digitalized [1–4]. The concordance rates in diagnosis between using conventional light microscope and WSI range from 87 to 96%, depending on various subspecialties [1, 5–9]. However, most of previously published studies mainly targeted small specimens and excluded cases requiring immunohistochemistry, such as lymphoma cases. So far there is only one published report investigating the concordance rate between digital pathology and conventional microscopic histopathology and the researchers found that WSI was a reliable technology for lymphoma diagnosis [10].

Follicular lymphoma (FL) is one of the most common low-grade B cell lymphomas. FL is a heterogeneous disease, and may be further divided into many different subtypes in terms of age of onset, involved organs and genetic abnormality. In the revised fourth edition of World Health Organization (WHO) classification of tumors of hematopoietic and lymphoid tissues, primary intestinal FL (PI-FL) is listed as a rare and specific variant of FL with distinct clinical and biological features [11]. PI-FL predominantly occurs in the second portion of duodenum as multiple whitish polypoid nodules [12, 13]. Unlike patients with systemic nodal FL, most patients with PI-FL have localized diseases and excellent prognosis even without treatment [11–13]. However, long-term follow-up is recommended as rare cases with histological transformation to high-grade lymphoma have been reported [14, 15]. Considering the uniqueness of PI-FL, thorough clinical, laboratory, endoscopic and radiologic workups to differentiate PI-FL from systemic nodal FL with secondary gastrointestinal (GI) tract involvement have been suggested [16].

PI-FL shares the same histopathology, immunophenotype, and the hallmark t(14;18) translocation with systemic nodal FL [12, 17]. On the other hand, expression of certain markers, such as memory B-cell marker CD27, molecules related to B-cell homing such as chemokine (C-C motif) ligand 20 (CCL20) and mucosal vascular addressin cell adhesion molecule 1 (MAdCAM-1), lack of activation-induced cytidine deaminase (AID), and variable immunoglobulin heavy chain gene (*VH*) usage suggest a genetic similarity of PI-FL with gastric mucosa-associated lymphoid tissue lymphoma, but different from systemic nodal FL [13, 17–20]. In addition, follicular dendritic cells (FDC) residing in the periphery of neoplastic follicles is more frequent in PI-FL cases as compared to nodal FLs, in which the FDC usually form dense meshworks. Takata K. et al. thus suggested that recognizing different FDC patterns is useful for differentiating PI-FL from secondary GI involvement by systemic FL [13].

It is important to make an accurate diagnosis by endoscopic biopsy specimens to facilitate appropriate management. In this study we retrospectively analyzed PI-FL cases in an attempt to identify morphological features of PI-FL by measuring various parameters on WSI. These morphological clues might be useful in differentiating reactive lymphoid hyperplasia from PI-FL in endoscopic biopsy specimens.

## Methods

### Patients

We searched our databases of the Department of Pathology and Laboratory Medicine at the Taichung Veterans General Hospital, Taichung, and Chi-Mei Medical Center, Tainan, Taiwan for PI-FL from January 2001 to December 2018. Cases of systemic nodal FL with secondary GI tract involvement were excluded after clinical investigation and imaging studies.

We reviewed medical records for clinical information, including age, gender, anatomical sites, clinical symptoms, endoscopic findings, sampling methods, and staging results according to the Lugano Classification. The Follicular Lymphoma International Prognostic Index (FLIPI) was used for the evaluation of disease status for prognosis [21]. Follow-up period was measured from the date of diagnosis to the date of last follow-up. For comparison, 17 endoscopic biopsy specimens from the corresponding GI sites diagnosed as reactive follicular hyperplasia were recruited as controls.

### Histopathologic and immunohistochemical analyses

The cases diagnosed as FL were confirmed by three experienced hematopathologists (RCW, STC and SSC) according to the 2017 WHO criteria. Two of us (HNL and RCW) confirmed the diagnosis of reactive hyperplasia of the control cases.

Supplementary Table 1 lists the antibodies used and the staining condition for immunohistochemistry. The staining pattern of FDC meshworks was classified either as nodal type/pattern (> 30% positive FDC cells with FDC mainly located in the center of the follicles) or duodenal type/pattern (< 5% positive FDC cells with FDC in the periphery of the follicles), as previously defined by Takata K. et al. [22]

### Interphase fluorescence in situ hybridization (FISH)

Locus-specific interphase FISH was performed on paraffin-embedded tissue sections of 4 μ thickness as previously described [23, 24]. In brief, de-paraffinized sections were pre-treated by pressure-cooking for 10 min in Milli-Q water and subsequent incubation in pepsin solution for 25 min at 37 °C to increase DNA accessibility. Sections were then dehydrated through ethanol and air-dried. The appropriate probe mix (1.0 μ l) was applied to the tissue section and covered with a round 8 mm cover slip. Both probe and target DNA were simultaneously denatured at 73 °C for 5 min and incubated up to 2 days at 45 °C. Post-hybridisation washes were performed according to the “rapid-wash protocol” provided by Vysis, Downers Grove, IL. Sections were counterstained with 4,6-diamidino-2-phenylindole (DAPI) and mounted in Vectashield antifade solution (Vector Laboratories, Burlingame, CA, USA).

Dual Color Break Apart Rearrangement probes for *IGH* and *BCL2* and Dual Color Dual Fusion Translocation probe for *IGH/BCL2* reciprocal translocation (Vysis/Abbott Laboratories Ltd., Maidenhead, UK.) were used.

### WSI and measurements

**Fig. 1:**
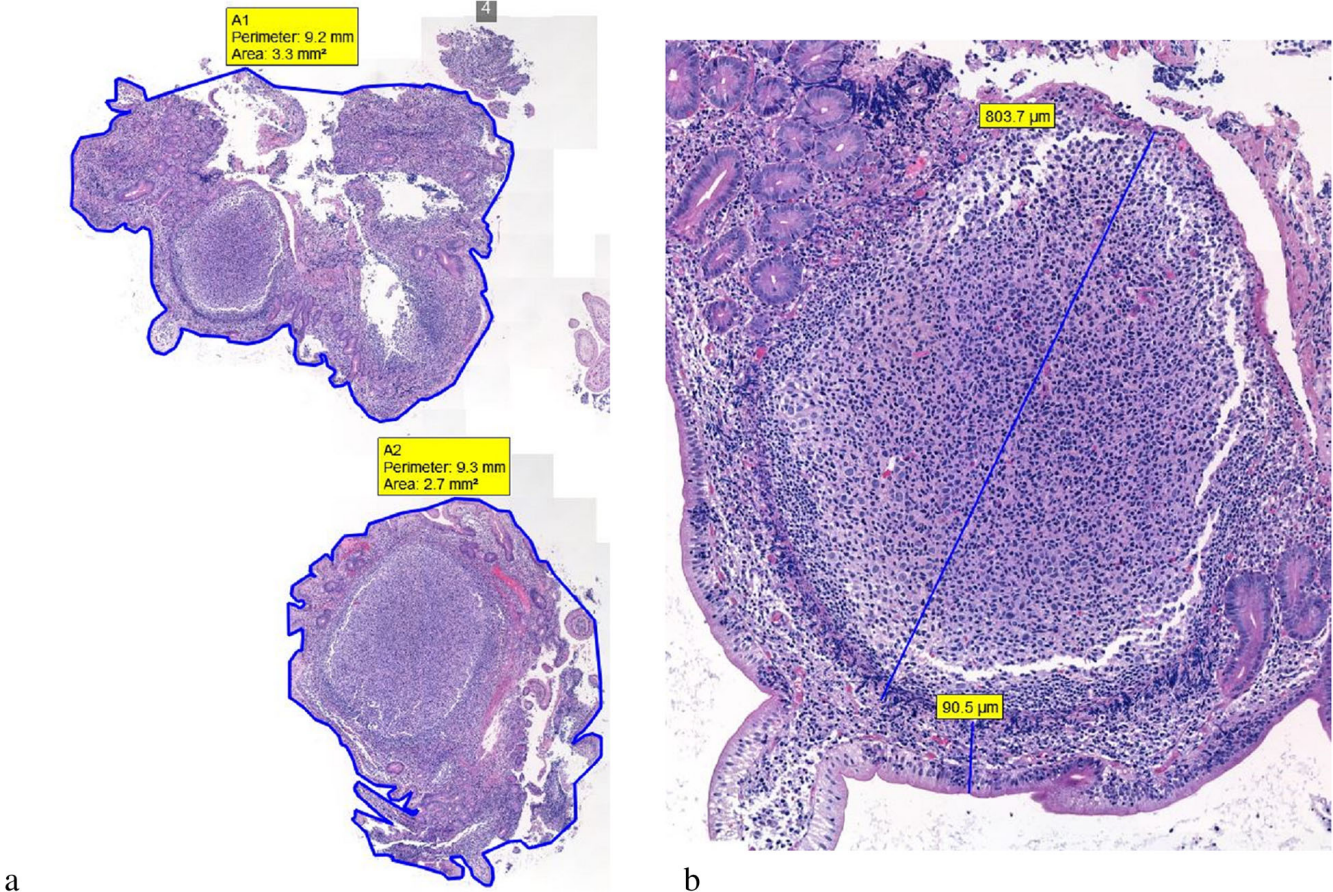
A representative example (Case no. 3) of using whole slide imaging in measuring various parameters. **a**, The density (D) of follicles is measured by dividing the number of lymphoid follicles by the total surface area of the biopsy specimen (with summation of the measurements from all the tissue fragments; D = number of follicles/area; in this case, 2/(3.3 + 2.7) = 0.3333). **b**, This panel shows the diameter (the greatest dimension) of a follicle (803.7 μm) and the nearest distance of this follicle to the surface epithelia (90.5 μm)

Hematoxylin and eosin-stained biopsy sections were scanned at 400x magnification (3D Histech Pannoramic 250, Budapest, Hungary). For each biopsy, the total surface area of the biopsy specimen was measured and the total number of follicles counted. For those specimens containing more than one fragment, the total number of follicles and the total areas of all fragments were respectively added up. We defined the density of follicles by dividing the number of lymphoid follicles by the total surface area of the biopsy specimen. The diameter (the greatest dimension) of a follicle (including the mantle zones and the follicular centers) and the shortest distance of that particular follicle to the surface epithelia were measured. Figure 1 depicts a representative example of various measurements using WSI. In this study we did not scan sections from resection specimens as the large number of follicles in such specimens may skew the data of endoscopic biopsies.

### Statistical analysis

The density and size of follicles and the shortest distance of the follicles to the surface epithelia were compared between the study and control groups. We used Mann-Whitney U test to obtain median values. Data analyses were performed using the Statistical Package for the Social Science (IBM SPSS version 22.0; International Business Machines Corp, New York, USA). Differences were considered significant when *P* < 0.05.

## Results

### Clinical features of the patients with PI-FL

Supplementary Table 2 lists the pertinent clinicopathological findings and follow-up information of the patients with PI-FL, while Table 1 summarizes the various clinical features of the PI-FL cases and the control cases with reactive hyperplasia. Eleven PI-FL patients were from Taichung Veterans General Hospital and ten patients were from Chi-Mei Medical Center. One of the cases from Chi-Mei Medical Center has been reported previously [25]. There was no difference in terms of age, gender, and biopsied sites between the patients with PI-FL and the controls.

**Table 1:** Pertinent clinical information of the patients with PI-FL and the control cases with reactive lymphoid hyperplasia

Parameters	PI-FL	Control
Case number	*n* = 21	*n* = 17
Age (years)		
Range	24–90	29–65
Median	61	54
Gender		
Male/Female	10/11	8/9
Symptoms		
None	8	15
Epigastric discomfort	4	2
Stool occult blood	2	0
Bowel habit change	1	0
Epigastric discomfort and bowel habit change	1	0
Abdominal pain	1	0
Recurrent post-prandial hiccup	1	0
Small bowel obstruction	1	0
NA	2	0
Site		
Duodenum	11 (52%)	11
Ileocecum	6 (29%)	4
Jejunum	2 (10%)	0
Colon, multiple	2 (10%)	2
Endoscopic findings		
Polypoid lesions	10	2
Whitish plaques	3	0
Nodularity	2	1
Hyperemia	1	6
Bloody fluid	1	0
NA	4	8
Sampling method		
Biopsy/Mucosal resection/Resection	16/1/4	17/0/0
Stage		
I/II/NA	13/5/3	
FLIPI		
0/1/2/3/NA	7/7/2/2/3	
Treatment		
None/CT/RT/NA	9/8/2/2	
Follow-up time (months)		
Range	12–208	
Median	41	

Abbreviation: *CT* Chemotherapy, *NA* Not available, *PI-FL* Primary intestinal follicular lymphoma, *RT* Radiotherapy

Of the PI-FL cases, there were 10 males and 11 females with a median age of 61 (range, 24–90). These diseases were detected either at routine medical check-up with endoscopy (*n* = 8) or when the patients sought medical advice with symptoms including epigastric discomfort (*n* = 5), stool occult blood (*n* = 2), bowel habit change (*n* = 2), abdominal pain (*n* = 1), recurrent post-prandial hiccup (n = 1) and small bowel obstruction (n = 1). The most common primary site was the duodenum (*n* = 11; 52%), followed by the ileocecum (*n* = 6; 29%), jejunum (n = 2; 10%) and multiple foci in the GI tract (n = 2; 10%). The endoscopic findings showed polypoid or nodular lesions in the presence or absence of ulceration, whitish plaques or hyperemia. Most cases (*n* = 16; 76%) were from endoscopic biopsies; with the remaining one (5%) from mucosal resection and four (19%) from bowel resection. Excluding the three cases with incomplete staging, the patients were either at stage I (*n* = 13) or II (*n* = 5). As shown in Table 1, around half of patients (*n* = 9) were followed without treatment (watch-and-wait), while the other half (*n* = 10) received localized radiotherapy or chemotherapy. No patient died of the disease in a median follow-up time of 41 months (range, 12–208).

### Histopathologic and immunohistochemical findings

**Fig. 2:**
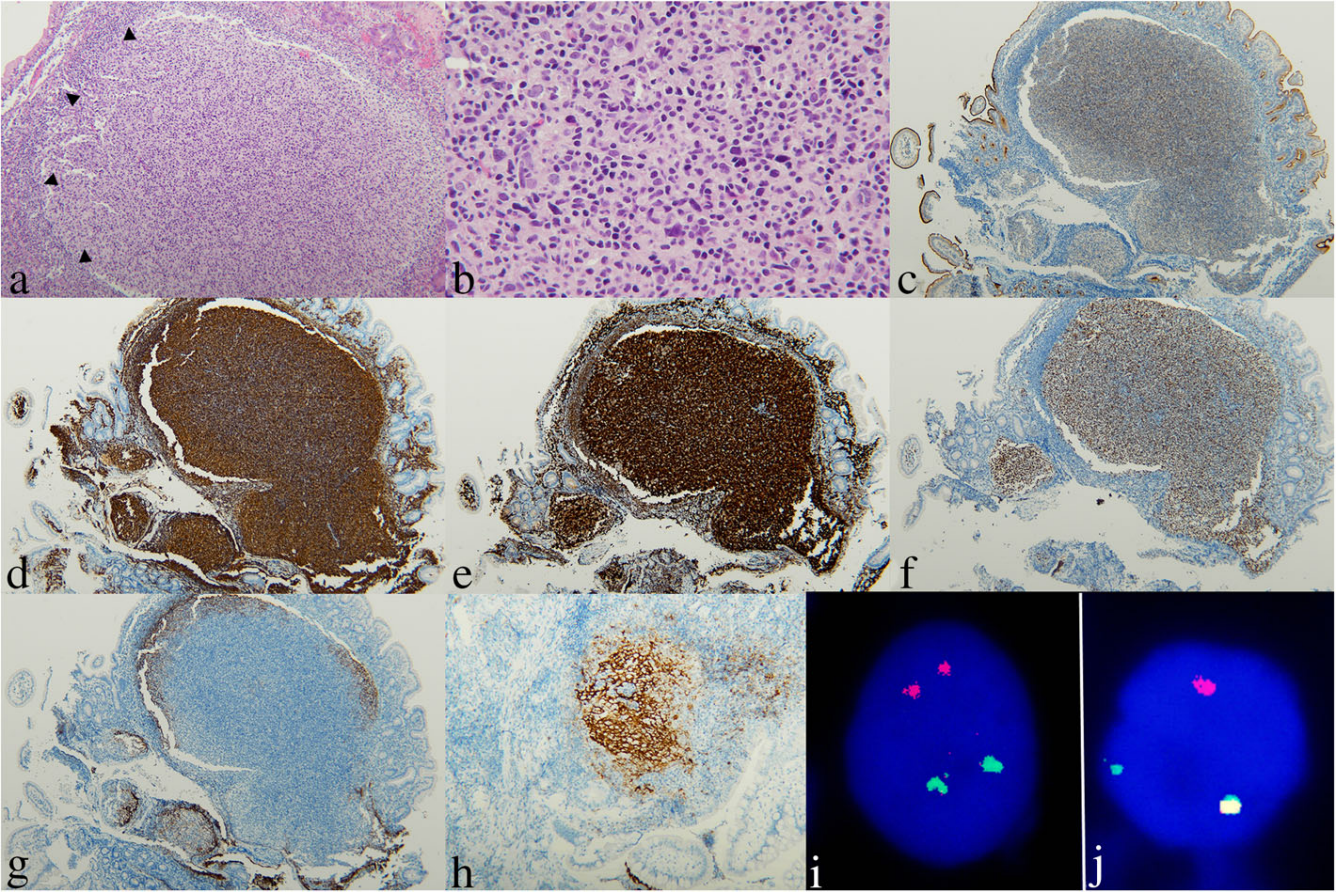
A representative case of a low-grade primary intestinal follicular lymphoma (Case No. 4, except Panel h, i and j). **a** Low-power view shows a large lymphoid follicle in the lamina propria of duodenal mucosa (H&E stain, × 40). The mantle zone indicated by black arrowheads is attenuated. In this case, the mucosa is intact, without erosion or ulceration. **b** The follicle is composed mainly of small- to medium-sized centrocytes without tingible body macrophages (× 400). Immunohistochemistry shows that the neoplastic cells express CD10 (**c**, × 40), CD20 (**d**, × 40), BCL-2 (**e**, × 40), and BCL-6 (**f**, × 40). The follicular dendritic meshworks of this case show a characteristic duodenal pattern by immunostaining with CD23 (**g**, × 40), in contrast to the nodal pattern in Case no. 11 (**h**, × 40). FISH assay using Vysis *IGH/BCL2* Dual Color, Dual Fusion Translocation Probe (*IGH* in SpectrumGreen and *BCL2* in SpectrumOrange). **i** A normal cell showing two orange and two green signals (2O2G), indicating no translocation of either *IGH* or *BCL2* genes. **j** A neoplastic cell with reciprocal *IGH/BCL2* translocation showing one orange, one green (representing the normal homolog) and one fused (orange/green) signal in yellow (1O1G1F; i and j from Case no. 21)

Table 2 summarizes the histological features and the immunohistochemical results. Microscopically, the neoplastic follicles were located in the lamina propria (Fig. 2a) with occasional surface erosion or ulceration. They were relatively round-shaped with absent or attenuated mantles and absence of polarity (Fig. 2a). Under high-power examination, the follicular center cells were monotonous comprising small- to medium-sized centrocytes without tangible body macrophages (Fig. 2b). All 21 cases were classified as low-grade FL.

**Table 2:** Histopathologic, immunohistochemical and FISH findings of PI-FL and control cases with reactive lymphoid hyperplasia

Parameters	PI-FL (n = 21)	Control (n = 17)
Features of the follicles		
Polarity	Lost	Preserved
Cellular components	Mainly centrocytes	Mixed populations
Tingible body macrophages	Absent	Present
Histologic grade		
1/2	19 (91%)/ 2 (10%)	NA
Immunohistochemistry		
CD10 expression	21 (100%)	
BCL6 expression	21 (100%)	
BCL2 expression	21 (100%)	
FDC pattern		Tight meshwork
Duodenal	14 (67%; 11 duodenum, 1 ileum and 2 colon)	
Nodal	6 (29%; 4 ileocecum and 2 jejunum)	
Mixed duodenal and nodal	1 (5%)	
FISH *IGH/BCL2*		
Positive/ Negative/ Failed/ Not tested	17/1/2/1	

Abbreviation: *FDC* Follicular dendritic cell, *FISH* Fluorescence in situ hybridization for reciprocal translocation of *IGH* and *BCL2* loci, *PI-FL* Primary intestinal follicular lymphoma

The neoplastic cells in all cases expressed CD20 (Fig. 2d) and BCL2 (Fig. 2e), with extrafollicular spreading to the overlying villi. The neoplastic follicular center cells of all cases expressed CD10 (Fig. 2c) and BCL-6 (Fig. 2f). FDC markers (CD21, CD23, or CD35) revealed a “duodenal pattern” in 14 cases, in which the FDC meshworks formed a condensed rim of enhanced staining at the periphery of the follicles but sparing the central area (Fig. 2g). The tumors of these 14 cases were located in the duodenum (*n* = 11), ileum (n = 1), and colon (*n* = 2). In six patients, the FDC pattern was nodal (Fig. 2h), and all these tumors were located in non-duodenal sites including ileocecum (*n* = 4) and jejunum (n = 2). Interestingly, in one case the FDC pattern in the initial biopsy was of duodenal type, while that of the resection specimen was of nodal type. Taken together, in our cases when the PI-FLs were located in the duodenum or other GI sites, FDC meshwork usually appeared as duodenal pattern; for those tumors occurring in the non-duodenal sites, the FDC meshworks tended to of nodal pattern. Coexisting duodenal and nodal patterns also occurred.

### Interphase FISH

FISH assay was successful in 18 cases. *IGH* and *BCL2* loci were rearranged in 94% (17/18) and 83% (15/18) cases, respectively. Seventeen (94%) cases showed *IGH/BCL2* reciprocal translocation.

### WSI analysis

Table 3 summaries the results of measurements using WSI between neoplastic follicles in PI-FL and control cases with reactive lymphoid hyperplasia. We found that the density of follicles was higher in PI-FL than that of reactive hyperplasia (median 0.5/mm^2^ vs. 0.2/mm^2^; *p* < 0.01) and that the neoplastic follicles were significantly larger (median diameter 756.9 μm vs. 479.7 μm; *p* < 0.01). The shortest distance of follicles from the surface epithelia was of no statistical significance between these two groups (104.7 μm vs. 177.8 μm; *p* = 0.056), although that of the PI-FL cases tended to be closer to the mucosal surface.

**Table 3:** Comparisons between neoplastic follicles in PI-FL and control cases with intestinal reactive lymphoid hyperplasia

	PI-FL (n = 17)	Control (n = 17)	*p* value
Diameter (μm)	756.9 (576.8–929.9)	479.7 (337.1–607.2)	0.001**
Distance from surface epithelia (μm)	104.7 (75.9–194.9)	177.8 (99.2–302.9)	0.056
Density (No./Surface area)	0.5 (0.3–0.7)	0.2 (0.1–0.3)	0.004**

Mann-Whitney U test. Median (Q1-Q3). *p < 0.05, **p < 0.01

Abbreviation: *No.* Number of follicles

## Discussion

Although rare, PI-FL has been characterized recently, particularly the duodenal-type FL. Surgical specimens are easier for diagnosis; however, with increasing use of endoscopy, accurate pathologic diagnosis on small biopsy specimens remains challenging. In this current study of PI-FL with WSI, we found that the average density and diameter of neoplastic follicles were larger than those of reactive lymphoid hyperplasia in the GI tract. Interestingly, in contrast to the study by Crocker et al in 1983, in which they used image analyzer and found that reactive lymph nodes had larger mean follicle areas than nodal FL [26]. The discrepancy between their and our studies may come from the different types of specimens/organs used for analysis, that is, large nodal tissue vs. endoscopic intestinal biopsy specimens. In addition, the neoplastic follicles in our PI-FL samples tended to be located more superficially, although not statistically significant. Occasional ulcerations or erosions of the surface mucosa were noted as well. Thus, we suggest that when encountering endoscopic biopsies with the presence of superficially located large follicles and a high density of follicles, immunohistochemistry is mandatory to exclude the possibility of PI-FL. Further WSI studies might include cases of systemic nodal FL with secondary involvement of GI tract, to compare the histologic parameters of neoplastic follicles between primary and secondary FL.

In keeping with other series of PI-FL [27, 28], the majority of our patients were middle-aged or elderly. One-third of our patients were asymptomatic and the remaining patients presented with ambiguous abdominal discomfort or bloody stools. In the literature, most of the cases were detected by endoscopy with typical polypoid lesions in the duodenum (65%), followed by jejunum and ileum (20%) [13]. Our cases showed a similar trend with duodenum as the most common site (11/21; 52%), followed by ileocecum and jejunum (8/21; 38%). In the largest series of PI-FL so far in the literature, Schmatz et al reported that in 11 (17%) of 63 patients another segment of the small bowel (i.e., jejunum and/or ileum) was involved [12]. In our study, in most of the cases (19/21; 91%), the tumors were located only in a single anatomic site. Primary FL at different GI sites has been reported to possess different endoscopic features as visualized by more advanced and detailed endoscopic examination. For example, the most frequent endoscope finding in the stomach was ulcerated submucosal tumor while small granular lesions were the predominant findings in the duodenum [29–31]. All of our cases showed low clinical stage without mortality or high-grade transformation during the follow-up period. Application of the FLIPI developed for nodal FL seemed irrelevant to the prognosis of our patients. However, data from more cases should be collected to investigate the feasibility of using FLIPI in PI-FL.

Immunophenotypically, the neoplastic follicular center cells expressed germinal center cell markers CD10 and BCL-6, with aberrant expression of BCL-2 due to t(14;18)/*IGH-BCL2* translocation. In our current study, all PI-FL cases demonstrated BCL-2 protein expression and all cases expressed CD10 and BCL-6. The characteristic duodenal pattern of FDC meshworks was observed in all of our duodenal examples of PI-FL, while those in the non-duodenal sites had either nodal or duodenal patterns. The phenomenon was also observed in a previous study [22]. Interestingly, one of our cases had FDC meshworks in a mixed nodal and duodenal pattern. To our knowledge, to date there are no data suggesting PI-FLs at different GI sites might have different prognosis. The significance of FDC patterns has not yet been fully investigated either.

Using FISH, we confirmed *IGH/BCL2* translocation in most (94%) of the successfully assayed cases, while the neoplastic cells in all cases expressed BCL-2 protein. The hallmark translocation t(14;18)(q32;q21) was detected in around 85% of PI-FL by FISH [12, 22], similar to that of nodal FL. Although in our previous study we found a relatively lower frequency (63%) of t(14;18) in low-grade FL in Taiwan [23]; in the current study we found that PI-FL in Taiwan carried a similar rate of t(14;18) as that of the Western nodal FL and Japanese PI-FL. The prognostic significance of t(14;18) in patients with PI-FL has not been delineated yet. Studies on larger numbers of cases are warranted.

## Conclusions

The incidence of PI-FL might be underestimated as the patients might be asymptomatic or might have ambiguous symptoms, as exemplified in our study that eight (38%) patients were discovered at health examination with endoscopy. Although the case number is limited, our study, so-far the largest from Taiwan, showed histologic and clinical features characteristic of PI-FL. Most importantly, our study is the first to utilize digital imaging technique to assess the histologic parameters of PI-FL vs. intestinal reactive lymphoid hyperplasia. Our results indicate that superficially located large follicles and a high density of follicles in endoscopic biopsy specimens are surrogates for PI-FL and immunohistochemistry is mandatory to confirm the diagnosis. Larger series of studies are required to better characterize and to verify the parameters measured from WSI and their usefulness as adjuncts for pathological diagnosis.

## Supplementary information

**Additional file 1: Table S1.** Antibodies used and the immunostaining conditions for this study. **Table S2.** Pertinent clinicopathological findings and follow-up information of the patients with primary intestinal follicular lymphoma.

## Data Availability

All data are available from the first author upon request.

## Abbreviations

FDC: Follicular dendritic cell
FISH: Fluorescence in situ hybridization
FL: Follicular lymphoma
FLIPI: Follicular Lymphoma International Prognostic Index
GI: Gastrointestinal
PI-FL: Primary intestinal follicular lymphoma
WHO: World Health Organization
WSI: Whole slide imaging
