# Supplementary material for: Density and size of lymphoid follicles are useful clues in differentiating primary intestinal follicular lymphoma from intestinal reactive lymphoid hyperplasia
Source: Diagn Pathol. 2020 Jul 7;15:82. doi: 10.1186/s13000-020-00991-3 (PMC7341590; doi:10.1186/s13000-020-00991-3)
Supplement: Supplementary file 1 — Additional file 1: Table S1. Antibodies used and the immunostaining conditions for this study. Table S2. Pertinent clinicopathological findings and follow-up information of the patients with primary intestinal follicular lymphoma. [file 13000_2020_991_MOESM1_ESM.docx]

**Supplementary Table 2.** Pertinent clinicopathological findings and follow-up information of the patients with primary intestinal follicular lymphoma.

| Case/Sex/Age | Clinical features | | | Bx./  Operation | Histologic  grading | Immunohistochemistry | | | | FISH *IGH/BCL2* | Stage | FLIPI | Tx | FU (m) |
| --- | --- | --- | --- | --- | --- | --- | --- | --- | --- | --- | --- | --- | --- | --- |
|  | Symptoms | Site | Endoscopic  findings |  |  | CD10 | BCL2 | BCL6 | FDC pattern |  |  |  |  |  |
| 1/F/62 | NA | Ileum | Polypoid | Bx. | 1 | + | + | + | DP | + | NA | NA | NA | AWD (0) |
| 2/F/75 | Epigastric discomfort | Cecum | Polypoid | Bx. | 1 | + | + | + | NP | + | IA | 3 | Nil | AWD (105) |
| 3/M/66 | Health exam | Duod. | Whitish plaques | Bx. | 1 | + | + | + | DP | + | IA | 3 | Nil | AWD (102) |
| 4/F/53 | Health exam | Duod. | Hyperemia | Bx. | 1 | + | + | + | DP | + | IA | 0 | Nil | AWD (12) |
| 5/M/59 | Health exam | Duod | Polypoid | Bx. | 1 | + | + | + | DP | + | IA | 1 | Nil | AWD (20) |
| 6/M/90 | Epigastric discomfort | Duod. | Whitish plaques | Bx. | 1 | + | + | + | DP | + | NA | NA | Nil | AWD (66) |
| 7/M/55 | Epigastric discomfort | Duod. | Whitish plaques | Bx. | 1 | + | + | + | DP | Failed | IA | 0 | Nil | AWD (33) |
| 8/M/63 | Health exam | Duod. | Polypoid | Bx. | 1 | + | + | + | DP | + | IA | 1 | Nil | AWD (47) |
| 9/F/66 | Health exam | Duod. | Polypoid | Bx. | 1 | + | + | + | DP | Not tested | IA | 2 | R-COP | AWD (41) |
| 10/F/64 | Epigastric discomfort | Duod. | Polypoid | Bx. | 1 | + | + | + | DP | + | IA | 1 | Nil | AWD (18) |
| 11/M/24 | NA | Ileum | Polypoid | Bx. | 1 | + | + | + | NP | + | NA | NA | NA | AWD(0) |
| 12/M/70 | Bowel habit change | A, T, & D colon | Multiple polyps | Bx. | 1 | + | + | + | DP | Failed | IA | 1 | Endoxan, RB, R maintenance | NED (105) |
| 13/F/65 | Bowel habit change, epigastric discomfort | Duod. | Multiple polypoid lesions in 2^nd^ & 3^rd^ portion | Bx. | 1 | + | + | + | DP | + | IB | 1 | R-COP | NED (62) |
| 14/F/39 | Recurrent hiccups for a month | Duod. | Uneven mucosa with polypoid change | Bx. | 1 | + | + | + | DP | + | IA | 0 | RT | NED (42) |
| 15/M/56 | Health exam | Duod. | Mucosal nodularity | Bx. | 2 | + | + | + | DP | + | IA | 0 | RT | NED (35) |
| 16/M/47 | Health exam | Ileocecum† | Multiple nodular lesions | Bx then r’t hemi. | 1 | + | + | + | DP (Bx) and NP (Res) | + | IIA | 0 | COP x6, CEOP x6 | NED (208) |
| 17/F/49 | Small bowel obstruction | Ileum |  | Seg resection | 1 | + | + | + | NP | - | IIA | 0 | Oral endoxan | NED (154) |
| 18/F/61 | Abdominal pain | Terminal ileum | Nil | R’t hemi | 1 | + | + | + | NP | + | IIEA | 1 | Nil | DOUD (25)‡ |
| 19/M/56 | Tarry stool with occult blood | Jejunum | Bloody fluid in terminal ileum | Seg resection | 1 | + | + | + | NP | + | IIEA | 1 | R-COP | AWD (94) |
| 20/F/89 | Tarry stool | Jejunum |  | Seg resection | 2 | + | + | + | NP | + | IE | 2 | R maintenance,  Gazyva | NED (32) |
| 21/F/44 | Health exam | S colon§ |  | EMR | 1 | + | + | + | DP | + | IIE | 0 | R-COP | NED (15) |

Abbreviation: A, T, D, S colon: ascending, transverse, descending, and sigmoid colon, respectively; AWD, alive with disease; Bx, biopsy; Bx, biopsy; CEOP, Cyclophosphamide+ Etoposide+ Oncovin/ Vincristine+Prednisone/prednisolone; DOUD, died of unrelated disease; Duod., duodenum; DP, duodenal pattern; EMR, endoscopic mucosal resection; FDC, follicular dendritic cell; FISH, fluorescence *in situ* hybridization for reciprocal translocation of *IGH* and *BCL2* loci; FU, follow-up period; m, months; R’t hemi, right hemicolectomy; NA, not available; NED, no evidence of disease; NP, nodal pattern; RT, radiotherapy; RB, rituximab and bendamustine; R-COP, rituximab, cyclophosphamide, oncovin, and prednisolone; Res, resection; Seg resection, segmental resection; Tx, treatment.

†Case no.16 showed FL in the terminal ileum with nodal involvement and was previously reported.^21^

‡Case no. 18 was found to have a brain tumor with an impression of malignant astrocytoma such as glioblastoma by MRI at 24 months after right hemicolecomy and she died of the progressive brain tumor 4 more months later.

§Case no. 21 had involvement of multiple foci of the gastrointestinal tract including stomach and colon, as confirmed by biopsies. PET-CT scans suggested ileal involvement, and abdominal CT scans revealed lymphadenopathy along the mesenteric arteries and gastroduodenal region.
